# Increase of calnexin gene dosage boosts the secretion of heterologous proteins by *Hansenula polymorpha*

**DOI:** 10.1111/j.1567-1364.2007.00271.x

**Published:** 2007-07-06

**Authors:** Jens Klabunde, Sebastian Kleebank, Michael Piontek, Cornelis P Hollenberg, Stephan Hellwig, Adelheid Degelmann

**Affiliations:** 1 ARTES Biotechnology GmbH Erkrath, Germany; 2 Institut für Mikrobiologie, Heinrich-Heine-Universität Düsseldorf Düsseldorf, Germany; 3 Fraunhofer Institut für Molekularbiologie und Angewandte Ökologie Aachen, Germany

**Keywords:** calnexin, *Hansenula polymorpha*, chaperone, heterologous protein expression, secretion

## Abstract

The type I membrane protein calnexin is a conserved key component of the quality control mechanism in the endoplasmic reticulum. It functions as a molecular chaperone that monitors the folding state of nascent polypeptides entering the endoplasmic reticulum. Calnexin also behaves as a lectin, as its chaperoning activity involves binding of oligosaccharide moieties present on newly imported glycoproteins. We isolated the calnexin gene (*HpCNE1*) from the methylotrophic yeast *Hansenula polymorpha*, and used *HpCNE1* expression plasmids for supertransformation of *H. polymorpha* strains secreting target proteins of biotechnological interest. The elevated dosage of *HpCNE1* enhanced secretion of the four proteins tested: three glycoproteins and one unglycosylated product. Secretion of bacterial alginate epimerase AlgE1 was increased threefold on average, and secretion of both human interferon-γ and fungal consensus phytase twofold. With phytase and AlgE1 this improvement was all the more remarkable, as the secretion level was already high in the original strains (g L^−1^ range). The same approach improved secretion of human serum albumin, which lacks N-linked glycans, about twofold. Glycosylation of the pro-MFα1 leader may account for the effect of calnexin in this case. Our results argue that cooverexpression of calnexin can serve as a generally applicable tool for enhancing the secretion of all types of heterologous protein by *H. polymorpha*.

## Introduction

Heterologous expression of commercially valuable proteins on an industrial scale requires the ability to achieve high yields if production of the target protein is to be cost-effective. Standard strategies for achieving this aim include optimization of the coding sequence, maximization of gene dosage, and the use of strong, inducible promoters for controlled transcription of the target gene.

In the methylotrophic yeast *Hansenula polymorpha*, target gene copy numbers of 40–50 are routinely attainable using a standardized transformation and culturing procedure that generates mitotically stable strains by chromosomal integration of expression plasmid multimers. Furthermore, the *H. polymorpha* expression system exploits a number of strong promoters derived from genes of the methanol utilization pathway (for review see Gellissen, 2000).

Secretion of the desired heterologous protein is generally the preferred mode of expression, as it facilitates downstream processing. Furthermore, our growing knowledge of the secretion process in fungi and higher eukaryotes makes it possible to manipulate the host cell's secretory pathway so as to improve the yield of recombinant proteins. In eukaryotic systems, the endoplasmic reticulum (ER) plays the central role in the secretory process. An N-terminal secretion signal peptide directs the newly synthesized protein into the lumen of the ER, where it encounters a class of ER-resident auxiliary proteins termed foldases and chaperones. Notable members of this group are the BiP family of Hsp70-like chaperones, the PDI family of protein disulfide isomerases, and the lectin chaperones calnexin and calreticulin. By direct physical interaction, these helpers promote the correct folding of newly synthesized polypeptides and ensure that misfolded chains or aggregates are removed from the ER (for recent reviews see Ellgaard & Helenius, 2003; van Anken & Braakman, 2005).

A number of studies have analyzed the effects of altering the levels of ER chaperone expression on heterologous protein secretion in fungal systems. The results show a strong dependence on the target protein. Thus, overexpression of BiP enhanced the secretion of bovine prochymosin (Harmsen *et al*., 1996) and hirudin (Kim *et al*., 2003), but had no effect on that of plant thaumatin (Harmsen *et al*., 1996) and actually reduced the expression of β-glucosidase (Smith *et al*., 2004) and manganese peroxidase (Conesa *et al*., 2002). Similarly, overexpression of PDI resulted in significantly enhanced secretion of human platelet-derived growth factor, acid phosphatase (Robinson *et al*., 1994), and recombinant human serum albumin (rHSA) (Bao & Fukuhara, 2001), but failed to improve export of human interleukin-1β (Bao & Fukuhara, 2001) and mammalian G-protein-coupled receptors (Butz *et al*., 2003).

Calnexin and its paralog calreticulin are chaperones involved in the folding and quality control of newly synthesized glycoproteins. Calnexin is a type I integral membrane protein, and calreticulin is a soluble protein found in the ER lumen. Both are thought to act as lectins that interact with N-linked Glc_1_Man_9_GlcNac_2_ oligosaccharides present on nascent glycoproteins (Ware *et al*., 1995) until the correct protein conformation is attained or until irreversibly misfolded proteins have been degraded (Ellgaard *et al*., 1999). In *Saccharomyces cerevisiae*, only the calnexin homolog Cne1p has been identified. Cne1p is similar in structure to mammalian calnexin, except that it lacks a cytoplasmic tail and does not bind calcium (Parlati *et al*., 1995). *Saccharomyces cerevisiae* calnexin has been shown to function as a molecular chaperone in a manner similar to mammalian calnexin (Xu *et al*., 2004).

Overexpression of calnexin together with a heterologous reporter protein has been achieved in mammalian cells, insect cells, and a fungal expression system. Cotransfection of calnexin together with subunits of the nicotinic acetylcholine receptor (AChR) into COS and HEK293 cells enhanced the surface expression of assembled AChR twofold (Chang *et al*., 1997). A 2.9-fold improvement in thrombopoietin secretion was observed by Chung *et al*. (2004) after cosupertransfection of recombinant CHO cells with calnexin and calreticulin. In insect cells, coexpression of calnexin resulted in increased levels of a functional serotonin transporter (Tate *et al*., 1999) and the Shaker potassium channel (Higgins *et al*., 2003). Secretion of a heterologous heme-binding peroxidase in *Aspergillus niger* was reported to be increased fourfold to fivefold, but only in the absence of heme supplementation (Conesa *et al*., 2002). If heme was added to the medium, cooverexpression of calnexin had no synergistic effect on the levels of peroxidase secretion. This finding was interpreted as indicating a specific function for calnexin in the incorporation of heme into the apoenzyme, and a role for calnexin in proper folding of hemoproteins during incorporation of the prosthetic group was also postulated.

As yet, few studies have addressed the role of calnexin in heterologous protein expression in yeast systems. In contrast to the examples mentioned above, deletion, rather than overexpression, of calnexin was reported to enhance secretion efficiency in *S. cerevisiae*. Thus, disruption of the *CNE1* gene caused a more than twofold increase in secretion of α_1_-antitrypsin (Parlati *et al*., 1995) and an unstable lysozyme (Arima *et al*., 1998). The authors attributed this effect to an absence of ER quality control-related degradation as a consequence of the absence of calnexin function.

In view of the widely varying effects reported up to now, we set out to evaluate the effect of calnexin overexpression on the secretion of various structurally divergent heterologous model proteins expressed in *H. polymorpha*. Four target proteins were analyzed, two of which are already secreted at high levels (g L^−1^ range) in the presence of the endogenous calnexin gene copy. Here we report that the secretion efficiency of all four was significantly increased by the introduction of extra copies of the calnexin gene.

## Materials and methods

### *Hansenula polymorpha* strains and vectors

All recombinant *H. polymorpha* strains used in this study are based on the uracil-auxotrophic (*ura3*) host strain RB11 (Zurek *et al*., 1996). Target genes were cloned into the expression vector pFPMT121 or a modified version thereof. pFPMT121, which is derived from pFMD-22a (Gellissen *et al*., 1991), contains the *FMD* (formate dehydrogenase) promoter and the *MOX* (methanol oxidase) terminator as transcriptional control elements for inserted genes. A map of pFPMT121 is shown in Degelmann *et al*. (2002).

Stable expression strains used for overexpression of calnexin are briefly described in the following. Strain IFNG.23-2 (kindly provided by Rhein Biotech) secretes human interferon-γ and contains *c*. 40 copies of the human gene (Degelmann *et al*., 2002). Strain HSA.56-1, which secretes human serum albumin (HSA), was constructed by amplifying the sequence encoding the mature form of HSA from human liver cDNA (Clontech), fusing the fragment to the coding sequence for the *S. cerevisiae* preproMFα1 secretion leader, inserting the fusion into pFPMT121, and transforming the construct into RB11 and selecting for stable expression strains (Degelmann, unpublished results). HSA.56-1 harbors about 20 copies of the HSA expression construct. Strain AlgE1.52-4 contains <5 copies of a gene encoding *Azotobacter vinelandii* alginate epimerase AlgE1 fused to preproMFα1 (Degelmann *et al*., 2004). Authentic bacterial AlgE1 has been described in detail previously (Ertesvag *et al*., 1995). Strain Conphys.3–68 (kindly provided by DSM Nutritional Products) secretes a synthetic fungal phytase (‘consensus phytase’) and contains >100 copies of the synthetic gene, including a phytase signal sequence (Mayer *et al*., 1999).

### Culture conditions

*Hansenula polymorpha* strains were grown in rich medium (YPD; 2% Bacto peptone, 1% yeast extract, 2% glucose) or minimal medium (0.17% Yeast Nitrogen Base without amino acids and ammonium sulfate, 0.5% ammonium sulfate, 2% glucose). Cells that had been supertransformed with the endogenous calnexin-encoding gene were selected in YPD medium containing phleomycin (40–60 μg mL^−1^, depending on the sensitivity of the individual host strain). The media used for small-scale expression culturing were based on rich or minimal medium, and contained 1–2% glycerol as the sole carbon source (YPG and YNBG, respectively). The exact compositions of expression media are indicated in the figure legends. The *Escherichia coli* strains DH10B (Gibco) and TOP10 (Invitrogen) were used for plasmid cloning, and bacterial clones were cultured in Luria Bertani medium supplemented with ampicillin (50 μg mL^−1^).

### Cloning of the *H. polymorpha* calnexin gene and construction of calnexin expression plasmids

**Fig. 1:**
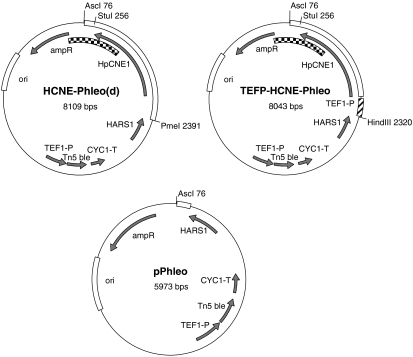
Maps of the *Hansenula polymorpha* calnexin expression plasmids HCNE-Phleo(d) and TEFP-HCNE-Phleo, and the control plasmid pPhleo. In HCNE-Phleo (d), the element designated HpCNE1 is delimited by AscI and PmeI sites and consists of the *HpCNE1* structural gene flanked by its natural upstream and downstream genomic sequences. In plasmid TEFP-HCNE-Phleo, the upstream flanking sequence is replaced by the *TEF1* promoter from *Arxula adeninivorans* (hatched bar). The checkered bar in HCNE-Phleo(d) and TEFP-HCNE-Phleo indicates the PCR fragment used to identify the plasmid-borne *HpCNE1* gene in supertransformants with increased target protein expression. HARS1 denotes an autonomously replicating sequence from *Hansenula polymorpha* (Roggenkamp *et al*., 1986). The phleomycin resistance marker present on all three plasmids consists of the structural gene Tn*5 ble*, the *TEF1* promoter (TEF1-P) and the *CYC1* terminator (CYC1-T) from *Saccharomyces cerevisiae* (Gatignol *et al*., 1990). The *ampR* and *ori* sequences are derived from the *Escherichia coli* vector pBR322.

On the basis of the known genomic sequence of *H. polymorpha* strain RB11 (Ramezani-Rad *et al*., 2003) the calnexin gene (*HpCNE1*), together with upstream and downstream regulating sequences, was amplified from genomic DNA of RB11 using the primer pair 5′-ATAGGCGCGCCCCAATGCTGGGCAGAAGAGATGAC and 5′-ATCGTTTAAACTGGAACCTACCTGACTCCGGAATG (HCNE-Asc and HCNE-Pme, respectively). The PCR fragment was cloned into pCR4Blunt-Topo (Invitrogen), and its sequence was verified. Replacement of the promoter and terminator sequences of vector pFPMT121 with the cloned *HpCNE1* fragment, and insertion of a phleomycin resistance marker (Gatignol *et al*., 1987), resulted in the calnexin expression plasmid HCNE-Phleo(d) (Fig. 1). In the second calnexin expression plasmid, TEFP-HCNE-Phleo, the endogenous upstream regulatory sequence of the *HpCNE1* cassette present in HCNE-Phleo(d) was replaced by the promoter sequence of the constitutively expressed *TEF1* gene from *Arxula adeninivorans* (Rösel & Kunze, 1995) (Fig. 1). The vector used for control transformations, pPhleo, was constructed by excising the *HpCNE1* fragment from HCNE-Phleo(d) with StuI/PmeI and religating the vector fragment (Fig. 1).

### Supertransformation and stabilization of *H. polymorpha* recombinant strains

Competent cells of the expression strains were prepared as described by Faber *et al*. (1994). The *HpCNE1* and control plasmids were transformed as circular DNAs into competent cells by electroporation in 2-mm cuvettes, using a Gene Pulser II (BioRad) at 1.5 kV, 200 Ω and 25 μF. After pulsing, 1 mL of YPD medium was added, and the cells were allowed to recover at 37°C for 4 h, and then plated on YPD/phleomycin. Transformant colonies were individually cultured in selective medium (YPD/phleomycin) for at least 20 generations to allow multiplication of plasmids, and this was followed by growth in nonselective medium (YPD) for another 20 generations to permit loss of nonintegrated plasmids (Gatzke *et al*., 1995). After a final selection step, the stabilized supertransformants were maintained on nonselective medium.

### Screening of supertransformants

Small-scale (3-mL) cultures of stabilized *HpCNE1* supertransformants were grown in YPG or YNBG (derepression medium) for 40–45 h at 37°C. Cells were removed by centrifugation, and the culture supernatants were assayed for the presence of secreted heterologous proteins.

rHSA was detected by a direct enzyme-linked immunosorbent assay (ELISA) procedure using peroxidase-conjugated antihuman albumin antibody raised in rabbit (Rockland). Detection of mannuronan C-5 epimerase AlgE1 and human interferon-γ was carried out by Western blotting (see below). Phytase was detected using either a phosphatase activity assay (Mayer *et al*., 1999) or sodium dodecyl sulfate polyacrylamide gel electrophoresis (SDS-PAGE) of culture supernatants.

### Diagnostic PCR and Southern analysis

Diagnostic PCR was performed using the primer 5′-GGCGACACGGAAATGTTGAATACTC-3′ (HCNE3), which hybridizes at the beginning of the *ampR* gene, and the primer 5′-GTCTCCAAGAAAGATTCCTAACCC-3′ (HCNE4), which anneals in the downstream half of the *HpCNE1* gene. The recovery of a PCR fragment of 0.87 kb thus indicates the presence of the expression plasmids HCNE-Phleo(d) or TEFP-HCNE-Phleo. The PCR was carried out using Taq Master Mix (Qiagen) and whole yeast cells as templates.

Genomic DNA for Southern analysis was prepared from yeast cells using the MasterPure Yeast DNA Purification Kit (Epicentre). The DNA fragment to be used as hybridization probe was amplified with HCNE-Phleo(d) as template and the primers HCNE-Asc and HCNE4. The purified fragment was labeled with fluorescein-11-dUTP using the Gene Images Random Prime Labeling Kit (GE Healthcare). Hybridized fragments were visualized by soaking the blotting membrane in DNA Thunder Chemiluminescence Reagent (Perkin Elmer Life Sciences) and exposing it to X-ray film.

### SDS-PAGE and Western blotting

SDS-PAGE of culture supernatants was performed under reducing and denaturing conditions using the Criterion System (BioRad) with 18% or 4–20% precast Tris-HCl gels. Gel lanes were loaded with 20-μL aliquots of untreated culture supernatant each, unless indicated otherwise. After electrophoresis, gels were either stained with Coomassie (GelCode Blue Stain Reagent, Pierce) or prepared for electroblotting. The proteins were transferred to nitrocellulose membrane using a Trans-Blot SD Semi-Dry Transfer Cell (BioRad). Blotted interferon-γ was reacted with goat anti-(human interferon-γ) (Sigma) as primary antibody, and rabbit anti-(goat IgG) (H+L) AP-conjugate (Biomol) as secondary antibody. AlgE1 epimerase was detected using rabbit anti-epimerase antibody (Hoidal *et al*., 2000) and goat anti-(rabbit IgG) AP-conjugate (BioRad). Antibody-decorated proteins were visualized by treatment with Nitro Blue tetrazolium/5-bromo-4-chloroindol-2-yl phosphate (NBT-BCIP) (Roche) staining reagent. Images of stained gels and Western blots were quantified using scion image beta 4.02 software (Scion Corporation).

### Other methods

Glycans were removed from secreted proteins by treatment of culture supernatants with endoglycosidase H (EndoH; New England Biolabs) under the conditions recommended by the manufacturer.

DNA manipulations, cloning and sequencing were carried out according to standard procedures (Sambrook & Russell, 2001).

## Results

### The *H. polymorpha* calnexin homolog

**Fig. 2:**
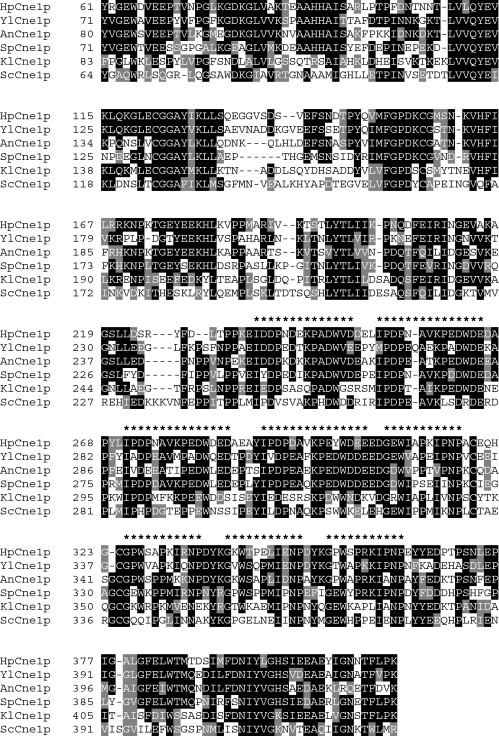
Multiple alignment of conserved domains of fungal calnexin proteins (Cne1p). Sequences were obtained from *Hansenula polymorpha* (Hp; this work), *Yarrowia lipolytica* (Yl; UniPROT ID Q9HFC6), *Kluyveromyces lactis* (Kl; UniPROT ID Q6CLT9), *Schizosaccharomyces pombe* (Sp; UniPROT ID P36581), *Aspergillus niger* (An; UniPROT ID Q8WZI9) and *Saccharomyces cerevisiae* (Sc; P27825). The alignment was generated with clustalw (http://www.ebi.ac.uk/clustalw/index) and shaded using boxshade 3.21 (http://www.ch.embnet.org/software/BOX_form.html). Identical amino acids are shaded in black, and conservative exchanges in gray. The two types of proline-rich repeats constituting the P-domain common to all known calnexins are indicated by asterisks. The nucleotide sequence of the *Hansenula polymorpha* calnexin gene has been deposited in the EMBL/GenBank/DDBJ databases under accession number AM409242.

Like other yeasts and fungi, *H. polymorpha* possesses a gene for calnexin but lacks a homolog of calreticulin. The calnexin gene encodes a protein of 557 amino acids with a molecular mass of 62 723 Da and a pI of 4.4. A stretch of 17 hydrophobic amino acids located 69 amino acids from the C-terminus indicates that, like all known calnexins, the *Hansenula* protein has a single transmembrane domain. The *H. polymorpha* calnexin sequence shares 29%, 34%, 44%, 47%, 48% and 33% overall identity with orthologs from *Saccharomyces cerevisiae*, *Kluyveromyces lactis*, *Schizosaccharomyces pombe*, *Yarrowia lipolytica*, *Aspergillus niger* and *Homo sapiens*, respectively. Thus, *HpCne1p* is more closely related to the *Aspergillus niger* and *Y. lipolytica* orthologs than to its *S. cerevisiae* counterpart. A multiple alignment of the central domains, including the conserved repeats, of fungal calnexins is shown in Fig. 2. The N-terminal and C-terminal regions of the various calnexins are widely divergent.

### Generation of *H. polymorpha* production strains that overexpress calnexin

The effect of calnexin overexpression was studied in recombinant *H. polymorpha* strains that produce heterologous proteins of biotechnological interest, some of them at g L^−1^ levels (see below). As calnexin is viewed as a classic lectin chaperone, we first focused on glycosylated proteins as reporters. The products known to undergo extensive N-glycosylation during passage along the *H. polymorpha* secretory pathway were fungal consensus phytase (Conphys), human interferon-γ (IFNG), and *Aztobacter vinelandii* C5-mannuronan (‘alginate’) epimerase, AlgE1. As a model protein that should not be subject to extensive posttranslational modification, we later chose HSA, which lacks N-glycosylation sites. The *H. polymorpha* strains that secrete these target proteins are designated Conphys.3-68, IFNG.23-2, AlgE1.52-4, and HSA.56-1, respectively. Each of the strains harbors multiple copies of the corresponding expression plasmid, integrated into the host genomic DNA in a tandem array. All of the heterologous genes are controlled by the potent and inducible *FMD* promoter and the *MOX* transcriptional terminator. The yields obtained from shake-flask cultures of IFNG.23-2 and HSA.56-1 were 5–10 mg L^−1^ of interferon-γ and 15–20 mg L^−1^ of HSA, respectively. In high-density fermentations, strain AlgE1.52-4 produced more than 1 g epimerase L^−1^ of culture medium (H. Sletta, pers. commun.). An optimized protocol for the fermentation of Conphys.3-68 resulted in the exceptionally high yield of 13.5 g phytase L^−1^ (Mayer *et al*., 1999). Indeed, this is the highest yield of a heterologous protein obtained from *H. polymorpha* so far.

To achieve overexpression of endogenous calnexin, each of these strains was supertransformed with the calnexin expression plasmids HCNE-Phleo(d) and TEFP-HCNE-Phleo and the negative control plasmid pPhleo (Fig. 1). We used phleomycin resistance to select for supertransformants, as the recipient strains are prototrophic. In our *H. polymorpha* transformation system, repeated cycles of growth under selective and nonselective conditions (passaging and stabilization) eventually result in the integration of multiple copies into the host cell genome. This strategy was employed to generate both the initial reporter strains and the stable supertransformants containing additional calnexin genes, controlled either by the cognate calnexin promoter or by the constitutive *TEF1* promoter of the yeast *A. adeninivorans*.

### Increased calnexin gene dosage enhances the secretion of heterologous glycoproteins

**Fig. 3:**
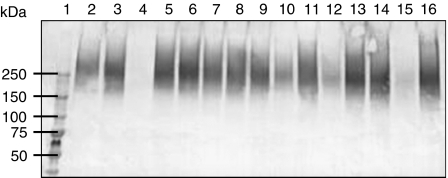
Secretion of AlgE1 from 14 randomly chosen AlgE1-HCNE supertransformants. Strains were cultured in YPG for 40 h at 37°C, and this was followed by SDS-PAGE and Western analysis of culture supernatants. Lane 1: protein size markers. Lane 2: parent strain AlgE1.52-4. Lanes 3–16: AlgE1-HCNE supertransformants.

**Fig. 4:**
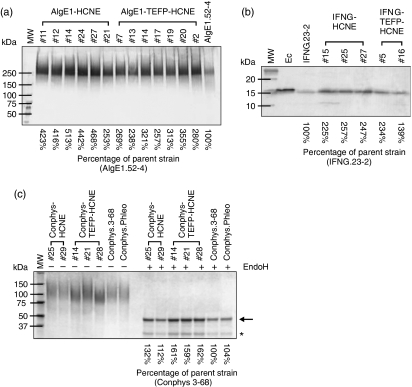
Secretion of target proteins by *Hansenula polymorpha* strains containing additional copies of the endogenous calnexin gene (*HpCNE1*). All strains were cultured in YPG. (a) Western blot of culture supernatants of AlgE1-HCNE and AlgE1-TEFP-HCNE supertransformants. (b) Western blot of EndoH-treated culture supernatants of IFNG-HCNE and IFNG-TEFP-HCNE supertransformants. The lane labeled Ec contains *Escherichia coli*-derived interferon-γ. (c) Coomassie-stained SDS-PAGE gel loaded with 1 : 4 diluted culture supernatants of Conphys-HCNE and Conphys-TEFP-HCNE supertransformants before and after EndoH treatment. The arrow indicates full-length 51-kDa phytase, and the star a proteolytic degradation product of phytase. The lanes show target proteins secreted by parent strains (AlgE1.52-4, IFNG.23-2, and Conphys.3-68), *HpCNE1* supertransformants (numbered lanes), and one control strain supertransformed with the plasmid pPhleo (Conphys-Phleo). Images were quantified by densitometric scanning as described in ‘Materials and methods’. The relative amount of product present in each lane is indicated as a percentage of that in the parent strain.

Stabilized *HpCNE1* supertransformants were cultivated in glycerol-containing media to switch on the *FMD* promoter, and, after 40–60 h at 37°C, the amounts of secreted product present in the culture supernatants were estimated and compared to those produced by the progenitor strains. Strikingly, we observed that at least one-third of the supertransformants analyzed secreted increased levels of product. This effect was especially obvious with AlgE1-HCNE supertransformants, where elevated product amounts were observed in more than 50% of cultures (Fig. 3). The bacterial C5-epimerase AlgE1 is a large protein with a calculated molecular mass of 147 kDa that lacks disulfide bonds (Ertesvag *et al*., 1995). *Hansenula*-specific hyperglycosylation of AlgE1 results in a heterodisperse product, which migrates as a typical smeared band in SDS-PAGE. Pronounced size heterogeneity due to glycosylation is also characteristic for *Hansenula*-derived interferon-γ and phytase (compare Fig. 4c).

The secretion efficiencies of selected AlgE1-HCNE, IFNG-HCNE and Conphys-HCNE supertransformants are compared to those of the corresponding precursor strains in Fig. 4a–c. We used densitometric analysis of the images shown in Fig. 4 to determine the relative amounts of secreted product (numbers for each lane shown below the images). The secretion levels of all three model proteins are clearly enhanced in *HpCNE1* supertransformants. The amounts of AlgE1 epimerase were increased 2.5-fold to 5-fold in individual AlgE1-HCNE supertransformants, as compared to the parent strain AlgE1.52-4. The culture supernatants of IFNG-HCNE and Conphys-HCNE supertransformants were deglycosylated with EndoH prior to electrophoretic fractionation to obtain clearly defined bands. Densitometric scanning of the images shown in Fig. 4b and c indicated improvement factors of 1.4–2.5 for interferon-γ and 1.1–1.6 for phytase.

### Increased calnexin gene dosage enhances secretion of an unglycosylated heterologous protein

**Fig. 5:**
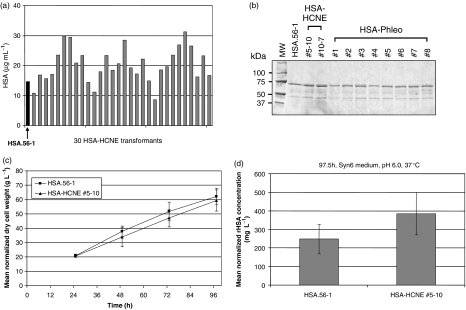
Secretion of rHSA by *HpCNE1* supertransformants and control strains. (a) Screening results obtained with 30 HSA-HCNE supertransformants (gray bars) as compared to the parent strain HSA.56-1 (black bar on the left). Strains were cultured in YNBG supplemented with 0.1 M sodium phosphate buffer (pH 6.0). Levels of rHSA present in culture supernatants were determined by direct ELISA. (b) Coomassie-stained culture supernatants from two HSA-HCNE supertransformant strains, eight randomly chosen control supertransformants containing the vector pPhleo (HSA-Phleo), and the parent strain HSA.56-1. Strains were cultured as above. (c, d) Summary of fedbatch-pro® fermentation results obtained with supertransformant HSA-HCNE #5-10 and the progenitor strain HSA.56-1. The data are based on four independent experiments performed in parallel with each strain. (c) Growth curves for 250-mL cultures of the supertransformant HSA-HCNE #5-10 and the parent strain HSA.56-1 in synthetic medium. The dry cell weights reached by the two strains at each time point are comparable. (d) The histograms depict the levels of secreted rHSA present in the growth medium after fermentation for the indicated time.

HSA, a protein of 66 kDa with 17 disulfide bonds, is efficiently secreted by various yeast hosts. HSA differs from the reporter proteins discussed in the previous section in that it lacks N-glycosylation sites. To test the effect of calnexin gene copy number on this presumably unglycosylated protein, we supertransformed the rHSA-producing *H. polymorpha* strain HSA.56-1 with the plasmid HCNE-Phleo(d). Screening of stabilized HSA-*HpCNE1* supertransformants by direct ELISA revealed 1.5-fold to 2-fold elevated levels of rHSA, relative to the control strain HSA.56-1, in almost 40% of culture supernatants (Fig. 5a). No increase was observed when HSA.56-1 was supertransformed with the negative control vector pPhleo. SDS-PAGE analysis of HSA.56-1 supertransformants confirmed these screening results (Fig. 5b). It should be noted that the increase in secretion efficiency observed with unglycosylated HSA may be due to the presence of the N-terminally linked secretion leader pro-MFα1, which is glycosylated in the *H. polymorpha* ER and only removed in a late Golgi compartment.

To investigate whether increased HSA expression is reproducible on a larger cultivation scale and under controlled cultivation conditions, one of the improved rHSA producers, strain HSA-HCNE #5–10, was selected for fed-batch fermentations on a 250-mL scale. The parent strain HSA.56-1 was fermented for the same time under identical conditions. Fed-batch fermentations were performed in synthetic medium, using a one-carbon source fermentation mode. Details of this work will be published elsewhere (Kleebank *et al*., in preparation). Briefly, the batch phase contained excess carbon source to allow rapid increase in biomass. At the end of the batch phase, feeding with glucose as the sole carbon source was started. Feeding was controlled by continuous monitoring of the dissolved oxygen concentration. Figure 5c and d summarize the results of four parallel fermentations performed with the fedbatch-pro® cultivation system (DASGIP). Under these conditions, derepression of the *FMD* promoter occurs, due to glucose limitation. The normalized fermentation data presented in Fig. 5c and d confirm the improvement in secretion capacity of HSA-HCNE #5-10 over the parental strain HSA.56-1. The average production yields of rHSA calculated for HSA.56-1 and HSA-HCNE #5-10 at nearly identical growth were 248 and 385 mg L^−1^, respectively, and were equivalent to a 50% increase in yield due to the presence of extra copies of the *HpCNE1* gene in transformant HSA-HCNE #5-10.

### Detection and copy number determination of integrated HpCNE expression plasmids

**Fig. 6:**
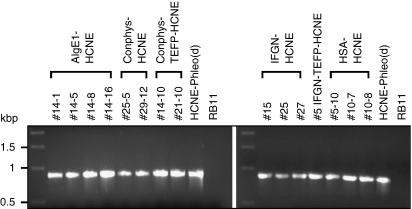
Detection of integrated *HpCNE1* expression plasmids in supertransformants that secrete increased amounts of target protein by PCR amplification of a diagnostic 0.87-kb fragment of HCNE-Phleo(d) and TEFP-HCNE-Phleo. The position of the amplified fragment is indicated in the plasmid maps shown in Fig. 1a and b. The two *HpCNE1* expression plasmids and the untransformed host strain RB11 are included as controls.

*HpCNE1* expression plasmids integrated into the genomic DNA of supertransformed strains were detected by PCR amplification of a specific plasmid fragment or by whole genome Southern hybridization. PCR amplification was performed using whole cells as the source of template, and the primers indicated in ‘Materials and methods’. These primers amplify a fragment comprising the downstream portion of the *HpCNE1* locus and the adjacent stretch of pBR322-specific plasmid sequence (see checkered bar in Fig. 1). Figure 6 shows that the 0.87-kb fragment specific for plasmids HCNE-Phleo(d) and TEFP-HCNE-Phleo is present in the supertransformed strains selected for increased secretion efficiency.

**Fig. 7:**
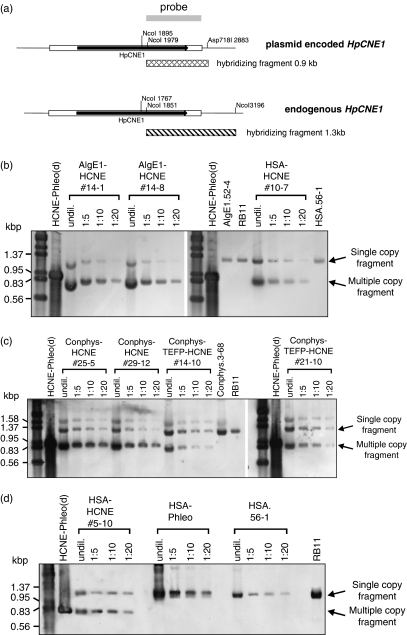
Copy number determination of integrated *HpCNE1* expression plasmids by semiquantitative Southern hybridization. NcoI/Asp718I-digested genomic DNA from each indicated strain was electrophoresed undiluted and in 1 : 5, 1 : 10 and 1 : 20 dilutions. The blot was hybridized with a fragment of the *HpCNE1* locus present on both expression plasmids. (a) Principle of the method (see also text for further details). The open bar and the black arrow represent the cloned *HpCNE1* segment and the *HpCNE1* structural gene, respectively. The shaded bars are explained in the diagram. (b–d) Analysis of *HpCNE1* supertransformant strains selected from those shown in Fig. 6. The dilutions of the genomic DNA of supertransformants are indicated above the lanes. The respective parent strains, one strain transformed with the ‘empty’ vector pPhleo [HAS-Phleo, (d)], the untransformed host strain RB11 and the *HpCNE1* expression plasmid HCNE-Phleo(d) are included as controls. The two labeled fragments identify the endogenous (single-copy fragment) and the plasmid-borne *HpCNE1* gene (multiple-copy fragment). The additional, larger fragment of about 1.6 kb seen in (c) most probably results from incomplete digestion of Conphys-HCNE genomic DNA.

We estimated the number of chromosomally integrated *HpCNE1* plasmids by semiquantitative Southern hybridization. The principle of the method is illustrated in Fig. 7a. A fragment from the distal part of the cloned *HpCNE1* locus was amplified by PCR and used as the hybridization probe. If the genomic DNA of a supertransformant is cleaved with suitable restriction enzymes, the probe will hybridize to two fragments that differ in size, one derived from the endogenous *HpCNE1* locus, and the other from the supertransformed calnexin plasmid. As illustrated in Fig. 7a, double digestion of genomic DNAs with NcoI–Asp718I results in labeled fragments that are cleanly separated by gel electrophoresis. Moreover, as the endogenous calnexin locus is present in only one copy per genome, it can serve as an internal reference for the estimation of the number of integrated calnexin plasmid copies. Comparison of the signal intensities of the single-copy fragment and the fragment derived from the supertransformed plasmid allows an estimation of plasmid copy number. The comparison is facilitated by the use of serial dilutions of the digested genomic DNA. Dilutions of 1 : 5, 1 : 10 and 1 : 20 were used for the Southern hybridizations.

Representative epimerase, phytase and rHSA producers were selected from those shown in Fig. 6 for copy number determination of integrated calnexin plasmids. The results are presented in Fig. 7b and c. Copy numbers are estimated by comparing the signal strength of the single-copy fragment in undiluted DNA with those of the plasmid-derived fragment in different dilutions, and identifying the closest match. From the images in Fig. 7b and c, plasmid-borne calnexin gene numbers of 5–10 in AlgE1-HCNE strains, ≤5 in Conphys-HCNE strains and <5 in HSA-HCNE strains can be inferred. The presence of integrated *HpCNE1* plasmids is compatible with the conclusion that the improved secretion efficiency observed in supertransformed strains is due to elevated expression of endogenous calnexin. It is, however, noteworthy that the ratio of supertransformed *HpCNE1* to target gene dosage is low in Conphys-HCNE (*c*. 100 phytase gene copies) and HSA-HCNE (*c*. 20 HSA gene copies) strains, whereas it is about equal in AlgE1-HCNE (<5 epimerase gene copies) strains.

## Discussion

In this article, we have provided evidence that increasing the dosage of the gene for the lectin chaperone calnexin in *H. polymorpha* enhances the secretion of various heterologous proteins of biotechnological interest. Three of these proteins, alginate epimerase AlgE1, human interferon-γ, and consensus phytase (Conphys), are N-glycosylated, and thus represent typical substrates for the action of calnexin. The highest relative increase in secretion level (fourfold) was observed in strains expressing the epimerase, whereas the improvement factors for interferon-γ and consensus phytase were 1.5–2.5 and 1.5–2.0, respectively. That an enhancement of yield was possible at all with the Conphys-HCNE strains is remarkable, as the parent strain itself, which harbors over 100 copies of the phytase gene, was thought to be at the limit of secretory capacity achievable for a heterologous protein (13.5 g L^−1^) (Mayer *et al*., 1999). The fourth heterologous protein investigated in this study, rHSA, is not susceptible to N-glycosylation. Nevertheless, this protein too is secreted more efficiently when *HpCNE1* is overexpressed in the same cell. The yield of extracellular rHSA was found to be increased by a factor of 1.5–2 when supertransformants were screened on a small scale (in 3-mL cultures) This degree of improvement was found to be reproducible under conditions of high-density fermentation on a 250-mL scale. Although the possibility cannot be ruled out that the propeptide of the *S. cerevisiae* mating factor α1, which serves as a secretion leader sequence for rHSA and is known to be N-glycosylated in *S. cerevisiae* as well as in *H. polymorpha*, is the actual substrate for *HpCNE1* activity, an interaction of calnexin with an unglycosylated protein such as rHSA would also be compatible with its postulated function as a classic molecular chaperone. There is increasing evidence that calnexin can perform chaperone functions by associating directly with the polypeptide backbone, in addition to binding to carbohydrate moieties. Thus, it was demonstrated that mammalian calnexin can associate with a protein substrate that completely lacks N-linked oligosaccharides *in vivo* (Danilczyk & Williams, 2001) and that a calnexin derivative lacking the lectin sites can nevertheless bind to, and prevent aggregation of, glycosylated substrates (Leach & Williams, 2004). Furthermore, the arm-like repeat domain, in addition to the lectin domains, was recently shown to be involved in the chaperone functions of Cne1p in *S. cerevisiae*. Investigation of mutant calnexin variants that specifically lacked either the repeat domain or the lectin domain revealed that Cne1p acts as a chaperone on unglycosylated as well as glycosylated substrates, and that both the lectin and the repeat domain are necessary for chaperone activity (Xu *et al*., 2004). Complex formation between calnexin and unglycosylated substrates has also been found in *Schizosaccharomyces pombe* after treatment of cells with tunicamycin (Beaulieu *et al*., 1999) or using naturally unglycosylated rHSA as a model protein (Marechal *et al*., 2004). In contrast to the results of the present study, raising the level of endogenous calnexin did not enhance secretion of rHSA in *Schizosaccharomyces pombe* (Marechal *et al*., 2004).

To achieve overexpression of endogenous calnexin in a given production strain, we increased the gene copy number by supertransformation, i.e. transformation of a recombinant host that already harbors multiple copies of the gene for a heterologous protein. Such a strain can, in principle, be transformed repeatedly, either with the previously introduced gene, or with a different gene that is to be coexpressed together with the first one. In *H. polymorpha*, the technique of sequential transformation has already been used successfully to increase the dosage of a single foreign gene (Mayer *et al*., 1999) and to achieve coexpression of two heterologous genes at relative levels that are optimal for the desired use of the final product (Janowicz *et al*., 1991; Gellissen *et al*., 1996). In a similar fashion, supertransformation can be employed to introduce extra copies of an endogenous gene that lead to an increase in its expression level in the host cell. It should be pointed out that the number of supertransformed gene copies present in stabilized integrants cannot be controlled, as integration is a stochastic process, and the number of plasmid copies ultimately integrated is not predictable. However, screening of sufficient numbers of supertransformants ensures that those showing the most favorable expression properties can be identified. When we followed this strategy in screening *HpCNE1* multicopy strains, it soon became obvious that a significant number (30–50%) secreted increased amounts of the target protein. The fact that no improvement was found in the remaining supertransformants analyzed can be attributed to the clonal variation that is routinely observed during the generation of *H. polymorpha* multicopy recombinant strains. Several factors may contribute to this variation. The supertransformants may express the target and *HpCNE1* genes at relative levels that preclude synergy. Alternatively, the plasmid-encoded *HpCNE1* genes might have integrated nonhomologously at chromosomal sites that prevent their expression, or homologously by single crossover into the chromosomal *HpCNE1* locus immediately after reaching the cell nucleus, which would maintain the single-copy status of *HpCNE1* in the supertransformed cell. In addition, a small proportion of supertransformants was always observed in which the secretion level of the target gene was significantly lower than that in the parent strain. These could result from remobilization of part of the originally integrated target gene plasmids. As the target gene plasmids and the *HpCNE1* plasmids have some sequences in common (HARS1, pBR322 backbone sequences), it is conceivable that homologous integration of *HpCNE1* plasmid multimers into the tandem array of target gene plasmids could induce the excision of a portion of the latter, effectively lowering the dosage of the target gene.

In control experiments, the production strains used for retransformation of *HpCNE1* were also transformed with plasmid pPhleo, which contained identical genetic elements, with the exception of the *HpCNE1* cassette itself. Here, increased secretion efficiency of target proteins was never observed. Together with the fact that multiple chromosomally integrated copies of exogenously introduced *HpCNE1* genes were detected in the improved strains, this strongly indicates that secretion enhancement is not due to genomic insertion or perturbance *per se*, but is a consequence of the increased dosage of the *HpCNE1* genes. It is reasonable to assume that the extra HpCNE1 genes are expressed, resulting in increased amounts of HpCne1p protein.

For supertransformation of production strains, we constructed two different *HpCNE1* expression cassettes. Both shared the authentic downstream region of the *HpCNE1* gene but differed in upstream control elements. The construct HCNE-Phleo(d) contained the authentic promoter region of *HpCNE1* to allow native transcriptional regulation of chaperone expression. In the second construct, TEFP-HCNE-Phleo, *HpCNE1* was placed under the control of the constitutive promoter of the *A. adeninivorans TEF1* gene (Rösel & Kunze, 1995). This promoter has been successfully employed for overexpression of various other host chaperones in the *H. polymorpha* system (Klabunde, 2003). Both types of regulatory elements resulted in clear improvement of reporter protein secretion, confirming the usefulness of each expression strategy.

Improvement of heterologous protein expression by increase of endogenous calnexin gene dosage has proven to be quite consistent in *H. polymorpha*. We observed positive effects on four heterologous proteins that differ widely in size and structure. For example, with a molecular mass of 147 kDa, AlgE1 is considerably larger than the rest of the target proteins studied in this work. It also lacks disulfide bonds, whereas phytase has five. HpCne1p is also capable of boosting the secretion efficiency of rHSA, a protein with 17 disulfide bonds that, in contrast to the remaining three, is not modified by N-glycosylation in *H. polymorpha*. Recently, we have evaluated the effects of increased *HpCNE1* gene dosage in strains producing two further unglycosylated proteins, hirudin and a fungal lipase. Like rHSA, these two proteins are targeted to the secretory pathway by the preproMFα1 secretion leader, and their extracellular yield was increased roughly two-fold in strains supertransformed with the plasmid HCNE-Phleo(d). Thus, the set of heterologous proteins that are favorably influenced by introducing multiple copies of *HpCNE1* has now been extended to six. In summary, the data presented in this article support the contention that overexpression of calnexin can augment the secretion efficiency of many, and perhaps all, stably expressed heterologous proteins in yeast and other fungal systems. Fine-tuning of calnexin overexpression and activity in a given recombinant yeast strain with regard to calibration of gene dosages and the best suitable promoter for calnexin expression thus holds promise as a reliable and reproducible method for improving the yield of secreted heterologous proteins in industrial production strains.

## Statement

Re-use of this article is permitted in accordance with the Creative Commons Deed, Attribution 2.5, which does not permit commercial exploitation.
